# Serum 25-hydroxy vitamin D level is associated with elastography-detected liver fibrosis in patients with type 2 diabetes mellitus in China

**DOI:** 10.3389/fendo.2024.1420088

**Published:** 2024-11-29

**Authors:** Nan Huang, Xianghui Su, Ting Yu, Xiaodong Wu, Bing Lu, Weixia Sun, Liqin Yao, Maoyun Wang, Yao Wang, Wenxuan Wu, Yingzhao Liu, Ting Yang, Ruidong Gao, Congqing Miao, Ling Li

**Affiliations:** ^1^ Department of Endocrinology, Zhongda Hospital, School of Medicine, Southeast University, Nanjing, China; ^2^ Institute of Glucose and Lipid Metabolism, Southeast University, Nanjing, China; ^3^ Department of Endocrinology, Changji Branch, First Affiliated Hospital of Xinjiang Medical University, Xinjiang, China; ^4^ Department of Gastroenterology, Zhongda Hospital, School of Medicine, Southeast University, Nanjing, China; ^5^ Department of Endocrinology, Kunshan Hospital Affiliated to Jiangsu University, Suzhou, China; ^6^ Department of Endocrinology, Yixing Hospital of Traditional Chinese Medicine, Yixing, China; ^7^ Department of Endocrinology, Baoying Hospital of Traditional Chinese Medicine, Yangzhou, China; ^8^ Department of Endocrinology, The Affiliated People’s Hospital of Jiangsu University, Zhenjiang, China; ^9^ Department of Endocrinology, Jiangdu People's Hospital Affiliated to Yangzhou University, Yangzhou, China

**Keywords:** type 2 diabetes mellitus, 25-hydroxy vitamin D, liver steatosis, liver fibrosis, body mass index

## Abstract

**Objective:**

In this cross-sectional study including patients with type 2 diabetes mellitus (T2DM) we aimed to explore the relationship between serum 25-hydroxy vitamin (25(OH)D) level and liver steatosis and fibrosis in the Chinese population.

**Methods:**

Patients visiting 16 clinical centers with T2DM were recruited. Their liver steatosis and fibrosis status were then assessed using elastography. Factors associated with steatosis and fibrosis were explored using regression analysis. Correlations between serum 25(OH)D levels and other patient characteristics were analyzed using linear regression.

**Results:**

In total, 1,513 patients with T2DM were included in the study. The prevalence of steatosis and fibrosis was 69.7%, and 34.6%, separately. A lower level of 25(OH)D was detected in patients with liver steatosis compared to those without, although it was not an independent predictor of this condition. However, 25(OH)D level was independently associated with liver fibrosis even when adjusted for age, sex, body mass index, hemoglobin A1c, insulin, and homeostatic model assessment of insulin resistance (OR = 0.964 [0.935-0.993], *P* = 0.015). When patients were separated into subgroups by sex, a correlation between 25(OH)D and fibrosis was identified in the male group (OR = 0.969 [0.940-0.998], *P* = 0.038).

**Conclusions:**

In conclusion, this multi-center, cross-sectional study in patients with T2DM showed that serum 25-hydroxy vitamin D level was strongly associated with liver fibrosis and this relationship was more pronounced in male patients.

**Clinical trial registration:**

https://clinicaltrials.gov/ct2/show/, identifier NCT05597709.

## Introduction

The prevalence of liver steatosis, with further development into nonalcoholic steatohepatitis (NASH) and fibrosis has been proven to be significantly higher in patients with type 2 diabetes mellitus (T2DM) (1, 2). The elucidation of risk factors and development of corresponding management strategies are therefore of great clinical importance.

Non-alcoholic fatty liver disease (NAFLD) and T2DM are closely associated (3). Patients with T2DM have been reported to have a higher prevalence of liver steatosis and advanced fibrosis (1, 4–6).

The causal relationship between diabetes and liver steatosis has not been thoroughly understood. Significant variability exists in the relationship between T2DM and liver steatosis, potentially due to the complicated pathological mechanisms (7). It has been reported that the prevalence of liver steatosis and the correlation between NAFLD and T2DM may be affected by ethnicity, race, and various other patient characteristics (8–10). The risk factors in T2DM that affect the development of liver steatosis have yet to be clarified.

Vitamin D plays a critical role in the regulation of the metabolism and endocrine and immune systems (11–13). Numerous studies have shown that vitamin D deficiency is associated with diabetes (14–16). More recent studies have also shown that vitamin D deficiency is among the risk factors for various diabetes complications (17–20). Although the overall effects need to be further confirmed, supplementation of vitamin D was suggested to facilitate the treatment of T2DM (19, 21). Vitamin D level may be also associated with liver steatosis. Previous studies have suggested that a higher level of serum 25-hydroxy vitamin D (25(OH)D) is associated with a lower risk of NAFLD (22, 23) and that vitamin D supplementation could improve the prognosis of chronic liver diseases (24). However, this is not certain and the association between liver steatosis and its progression still needs to be confirmed (25, 26).

In this multi-center, cross-sectional study, we aimed to examine the correlation between serum 25(OH)D levels and liver steatosis and fibrosis in patients with T2DM from the Chinese population.

## Methods

### Study design and population

An observational study aiming to assess the prevalence and development of liver steatosis/fibrosis in patients with T2DM was conducted in 16 clinical centers in central south China. The inclusion criteria included patients visiting these centers with T2DM from November 2022. The exclusion criteria included being enrolled in another trial, acute diabetes complications, active and serious infection, pregnancy, cancer, and mental disorders. The patient’s liver steatosis and fibrosis status were assessed using elastography. Clinical characteristics along with laboratory examination results were also collected. A total of 1,513 patients with T2DM were included in the study.

In this cross-sectional study, the characteristics of the patients recruited were compared between those with different liver steatosis/fibrosis statuses. Associations between serum 25(OH)D level with liver steatosis/fibrosis were specifically explored.

Elastography is a non-invasive imaging technique used to assess liver stiffness. It employs an ultrasound transducer to generate and capture shear waves, which propagate through the liver tissue. The speed of these shear waves is directly related to the stiffness of the liver, which in turn correlates with the degree of fibrosis. A higher shear wave velocity indicates increased liver stiffness and more advanced fibrosis. This method is widely accepted as a reliable tool for staging liver fibrosis in chronic liver diseases, including NAFLD and other conditions associated with fibrosis.

### Data collection

Liver steatosis was defined as a controlled attenuation parameter (CAP) score ≥244 db/m. Fibrosis was defined as a liver stiffness measurement (LSM) score ≥7.85 kPa.

Demographic characteristics, clinical information of diabetes status, serum lipids profiles, and inflammation markers in a blood test were collected. Serum 25(OH)D level was measured using high-performance liquid chromatography-tandem mass spectrometry (HPLC-MS/MS) in the clinical laboratory of Zhongda Hospital.

### Statistical analysis

Characteristics were compared between patients with liver steatosis or fibrosis and those without these conditions. Factors associated with steatosis/fibrosis were explored using regression analysis. In addition, correlations between serum 25(OH)D levels and other patient characteristics were analyzed using Spearman’s correlation analysis and logistic regression analysis. Normally distributed continuous data were presented as mean ± SD, while skew-distributed data were presented as median (range). Categorical data were presented as frequency (percentage). Differences in normally distributed data between two groups were analyzed using Student’s t-test. Skew-distributed data were compared using the non-parameter Mann–Whitney U test. Differences in categorical data were compared using the Chi-squared test or Fisher’s exact test. The correlated factors for liver steatosis and fibrosis were analyzed using single and multivariate logistic regression analyses. SPSS Statistics version 23.0 (IBM, Armonk, NY, USA) were used for statistical analysis. All tests were two-sided. *P* < 0.05 was considered = significantly significant.

## Results

### Characteristics of patients

In total, 1,513 T2DM patients were included in the study period. In 1,507 patients with valid liver steatosis data, 1,049 were identified with liver steatosis, with a prevalence of 69.7%. A total of 520 patients out of 1,509 with valid data were diagnosed with liver fibrosis, with a prevalence of 34.6%. The mean age for the individuals in this study was 58 ± 12.7 years old.

### Steatosis/fibrosis patients had lower serum 25-hydroxy vitamin D levels

To explore the potential risk factors for liver steatosis and fibrosis of patients with T2DM, comparisons with various characteristics were performed. As shown in **Table 1**, patients with steatosis showed significant differences from those without steatosis, including younger age, a higher prevalence of women, higher body mass index (BMI), a higher percentage of overweight/obesity, and higher insulin resistance but lower hemoglobin A1c (HbA1c) and shorter diabetes duration. Laboratory tests showed that the patients in the two groups had significant differences in serum lipid profile with higher levels of triglyceride (TG), low-density lipoprotein (LDL), and very low-density lipoprotein (VLDL), and lower levels of high-density lipoprotein (HDL) in the patients with steatosis. In addition, mild but statistically significant lower levels of 25(OH)D were detected in patients with steatosis compared to those without (Median 21.7 vs 20.3 nmol/L, *P* = 0.025).

**Table 1:** Comparison of characteristics between patients with T2DM with and without liver steatosis.

	Non-steatosis (n= 458)	Steatosis (n=1049)	*P-*value
Demographic			
Age (Year)	60.00 ± 12.40	56.50 ± 14.00	<0.001
Sex			0.021
Female	35.80%	42.10%	
Male	64.20%	57.90%	
Diabetes parameters			
Disease duration (Year)	10.00 (4.00-15.00)	6.00 (1.00-10.00)	<0.001
HbA1c (mmol/L)	9.51 ± 2.56	9.09 ± 2.14	0.002
Insulin-F (UIU)	6.09 (2.17-11.2)	8.51 (3.74-14.6)	<0.001
HOMA-IR	2.32 (0.75-4.14)	3.16 (1.34-5.45)	<0.001
Body weight and serum lipid profile			
BMI (kg/m²)	22.90 ± 2.88	26.00 ± 4.04	<0.001
BMI category			<0.001
Normal	53.00%	19.00%	
Overweight	40.40%	52.60%	
Obese	6.50%	28.40%	
TG (mmol/L)	1.19 (0.87-1.81)	1.72 (1.18-2.68)	<0.001
LDL (mmol/L)	2.34 (1.83-3.03)	2.50 (1.94-3.12)	0.011
HDL (mmol/L)	1.23 (1.00-1.50)	1.14 (0.94-1.36)	<0.001
VLDL (mmol/L)	0.66 (0.39-2.07)	1.27 (0.52-1.42)	<0.001
Cholesterol (mmol/L)	4.40 ± 1.24	4.63 ± 1.28	0.002
25-hydroxy vitamin D (nmol/L)	21.70 (16.80-27.90)	20.30 (15.60-25.60)	0.025

BMI, body mass index; TG, triglyceride; LDL, low-density lipoprotein; HDL, high-density lipoprotein; ALT, alanine transaminase; AST, aspartate transaminase; GGT, gamma glutamyl transferase; eGFR, estimated glomerular filtration rate; FT3, free triiodothyronine; FT4, free thyroxine; TSH, thyroid-stimulating hormone; hsCRP, high-sensitive C-reactive protein; UACR, urinary albumin to creatinine ratio.

Data are presented as mean (SD), median (25th-75th percentile [P25-P75]), or n and percentage. The *P-*values are calculated using Student’s t-test, the Kruskal–Wallis test, or the chi-squared test. HbA1c, hemoglobin A1C; HOMA-IR, homeostatic model assessment of insulin resistance; BMI, body mass index; TG, triglyceride; LDL, low-density lipoprotein; HDL, high-density lipoprotein; VLDL, very low-density lipoprotein.

When characteristics were compared between patients with and without fibrosis (**Table 2**), differences in age, gender, and HbA1c were not significant, while differences in BMI and lipid profiles were similar as in the steatosis analysis. Notably, patients with liver fibrosis also had significantly lower levels of serum 25(OH)D compared to those without fibrosis (Median 21.0 vs 19.3 nmol/L, *P* = 0.030).

**Table 2:** Comparison of characteristics between patients with T2DM with and without liver fibrosis.

	Non-fibrosis (n= 989)	Fibrosis (n=520)	*P*-value
Demographic			
Age (Year)	57.30 ± 13.30	58.10 ± 14.30	0.366
Sex			0.117
Female	38.70%	42.90%	
Male	61.30%	57.10%	
Diabetes parameters			
Disease duration (Year)	8.00 (2.00-13.00)	5.50 (1.00-11.80)	<0.001
HbA1c (mmol/L)	9.27 ± 2.28	9.11 ± 2.29	0.220
Insulin-F (UIU)	6.73 (2.69-12.30)	9.56 (4.72-15.70)	<0.001
HOMA-IR	2.49 (0.96-5.07)	3.34 (1.54-5.51)	<0.001
Body weight and serum lipid profile			
BMI (kg/m²)	24.40 ± 3.15	26.40 ± 4.04	<0.001
BMI category			<0.001
Normal	34.40%	19.80%	
Overweight	50.30%	46.10%	
Obese	15.30%	34.10%	
Waist-to-hip ratio	0.94 (0.90-0.97)	0.95 (0.91-0.99)	<0.001
Visceral fat area (cm^2^)	84.00 ± 33.00	102.80 ± 35.00	<0.001
Subcutaneous fat area (cm^2^)	169.20 ± 48.70	197.80 ± 61.80	<0.001
TG (mmol/L)	1.44 (0.99-2.33)	1.65 (1.18-2.60)	<0.001
LDL (mmol/L)	2.49 (1.94-3.13)	2.39 (1.85-3.05)	0.038
HDL (mmol/L)	1.19 (0.99-1.42)	1.11 (0.92-1.35)	<0.001
VLDL (mmol/L)	0.95 (0.44-1.91)	1.14 (0.45-1.54)	0.279
Cholesterol (mmol/L)	4.60 ± 1.27	4.48 ± 1.28	0.095
Alkaline phosphatase (U/L)	77.00 (62.00-95.80)	81.20 (65.00-99.90)	<0.001
Bile acid (μmol/L)	2.93 (2.10-4.45)	3.56 (2.50-5.90)	<0.001
25-hydroxy vitamin D (nmol/L)	21.00 (16.40-26.70)	19.30 (15.00-25.50)	0.030

Data are presented as mean (SD), median (25th-75th percentile [P25-P75]), or n and percentage. The *P-*values are calculated using Student’s t-test, the Kruskal–Wallis test, or the chi-squared test. HbA1c, hemoglobin A1C; HOMA-IR, homeostatic model assessment of insulin resistance; BMI, body mass index; TG, triglyceride; LDL, low-density lipoprotein; HDL, high-density lipoprotein; VLDL, very low-density lipoprotein.

### Multivariate analysis identified serum 25-hydroxy vitamin D to be independently correlated with liver fibrosis

To further explore the potential risk factors for liver steatosis and fibrosis in patients with T2DM, multiple logistic regression analysis was performed. The results indicated that multiple factors were independently associated with higher risks of liver steatosis and fibrosis, including female sex, a higher BMI, and shorter disease duration. However, age, HbA1c, insulin, and HOMA-IR were no longer associated with liver steatosis and fibrosis in the multivariate analysis. In addition, the correlation of some other characteristics, such as lipid profiles, was only significant with liver steatosis but not liver fibrosis. These results suggested that simple differences in these factors between the patient groups did not indicate close associations.

Considering the subtle differences in 25(OH)D between the patient groups, it was expected that its associations with liver steatosis and fibrosis may also be superficial. However, somewhat surprisingly, the results of the multivariate regression analysis (**Table 3**) showed that serum 25(OH)D level, although not an independent predictor of liver steatosis, was independently associated with liver fibrosis (OR = 0.964 [0.935-0.993], *P* = 0.015) even when adjusted for age, sex, BMI, HbA1c, insulin, and HOMA-IR. It also demonstrated that for every one standard deviation increase in 25(OH)D level, the risk of liver fibrosis was reduced by 3.60%. These results indicated that, although at a very mild level, differences in serum 25(OH)D could be a significant factor related to liver fibrosis in patients with T2DM.

**Table 3:** Multivariate analysis of the correlation between 25-hydroxy vitamin D with liver steatosis and fibrosis in patients with T2DM.

	Adjusted for age and sex		Adjusted for age, sex, BMI, HbA1c, insulin, and HOMA-IR	
Variable	OR (95% CI) model 1	*P* value	OR (95% CI) model 2	*P* value
Liver steatosis				
25(OH)D	0.982 (0.962, 1.003)	0.090	0.977 (0.948, 1.006)	0.113
Liver fibrosis				
25(OH)D	0.973 (0.952, 0.995)	0.010	0.964 (0.935, 0.993)	0.015

Results were obtained with logistic regression and given as OR and 95% CI. 25(OH)D, 25-hydroxy vitamin D.

### The association between serum 25-hydroxy vitamin D with liver fibrosis was specific for male patients

To further explore the relationship between 25(OH)D and liver fibrosis, we analyzed the correlations of serum 25(OH)D with other characteristics of patients with T2DM. As shown in **Table 4**, the multivariate linear regression analysis found that 25(OH)D was significantly correlated with age and sex, but not HbA1c, BMI, or high-sensitive C-reactive protein (hsCRP). Since BMI is the most significant associated factor for liver steatosis and fibrosis and female sex was surprisingly found to be associated with higher risk of liver steatosis and fibrosis in this study, we further explored the association between serum 25(OH)D and fibrosis in different BMI and gender subgroups. As shown in **Table 5**, a significant correlation between 25(OH)D and fibrosis could not be detected in patients separated into lean and overweight subgroups. These results may be explained by the reduced sample size in each subgroup and mild associations. However, when patients were separated into subgroups of different sexes, although not detected in the female group, a clear independent correlation between 25(OH)D and fibrosis was identified in the male group (OR = 0.969 [0.940-0.998], *P* = 0.038). It suggested that for every one standard deviation increase in 25(OH)D level, the risk of liver fibrosis was reduced by 3.10% in the male group. Patients with higher levels of 25(OH)D appeared to be at lower risk of liver fibrosis.

**Table 4:** Multivariate analysis of correlations between 25-hydroxy vitamin D and other characteristics of T2DM patients.

Variable	Coefficient	*P* value
Age	0.123	0.001
Sex	0.371	0.001
HbA1c	-0.376	0.056
BMI	0.026	0.835
hsCRP	-0.026	0.105

HbA1c, hemoglobin A1C; BMI, body mass index; hsCRP, high-sensitive C-reactive protein.

**Table 5:** Correlations between 25-hydroxy vitamin D and liver fibrosis in different subpopulations of patients with T2DM.

	Adjusted for age		Adjusted for age and BMI	
	OR (95% CI)	*P* value	OR (95% CI)	*P* value
Male	0.970 (0.944, 0.998)	0.035	0.969 (0.940, 0.998)	0.038
Female	0.979 (0.946, 1.014)	0.240	0.974 (0.939, 1.011)	0.174
	Adjusted to age		Adjusted to age and sex	
	OR (95% CI)	*P* value	OR (95% CI)	*P* value
Lean	0.959 (0.913, 1.008)	0.097	0.953 (0.906, 1.004)	0.068
Overweight	0.979 (0.957, 1.003)	0.085	0.978 (0.954, 1.002)	0.077

Results were obtained with logistic regression and given as OR and 95% CI. 25(OH)D, 25-hydroxy vitamin D.

## Discussion

In this cross-sectional study exploring the characteristics and potential risk factors for liver steatosis and fibrosis, we found that there was a high prevalence of liver steatosis and fibrosis among the patients with T2DM. The potential risk factors for liver steatosis/fibrosis included BMI, sex, and diabetes duration. Lower serum 25(OH)D levels were detected in patients with liver steatosis and fibrosis. Furthermore, serum 25(OH)D levels appeared to be independently associated with fibrosis and have more influence in male compared to female patients.

In this study, patients with T2DM visiting 16 centers, from community to tertiary hospitals in Jiangsu Province, located in the central China region, were studied. The prevalence of liver steatosis and fibrosis was comparable to previous studies carried out in the USA (5, 6), France (1), and other populations in China (27, 28). Therefore, a high prevalence of liver steatosis and fibrosis appears to be not highly associated with ethnicity.

A higher BMI was found to be the most significant risk factor for liver steatosis and fibrosis in this study, which was consistent with previous studies (29, 30). In contrast, a patient’s diabetes status was not significantly associated with liver steatosis and fibrosis. These results further indicate the complexity of the interaction between T2DM and liver steatosis, which involves crosstalk among lipid and glucose metabolism, as well as inflammation (31–34). In this study, age did not appear to be strongly associated with a risk of steatosis or fibrosis. Previous studies about a correlation between age and NAFLD did not produce consistent results (4, 35, 36). Furthermore, in the T2DM population, NAFLD risk may decrease with increasing age (37). These results indicated that age may not be among the significant risk factors for liver steatosis and fibrosis. Interestingly, BMI did not play a role in the relationship between vitamin D and liver fibrosis in this study. This could be attributed to the diverse population sampled, which encompassed individuals with various stages of T2DM from 16 medical centers, rather than focusing on specific conditions such as obesity, liver steatosis, or liver fibrosis. As a result, there may have been a significant number of participants with less severe liver disease and better weight management.

Although the differences in serum 25(OH)D levels appeared to be subtle, the correlation between 25(OH)D with liver fibrosis was statistically significant even when adjusted for multiple other risk factors. The 25(OH)D levels detected in this study were at the edge of 25(OH)D deficiency (20 nmol/L). It has long been known that patients with T2DM are at more risk of 25(OH)D deficiency (14–16) therefore the results were not surprising. What was surprising is that the multivariate regression analysis found a non-significant relationship between 25(OH)D levels and liver steatosis, but a significant correlation with liver fibrosis in this study. There are two possible reasons. One is that 25(OH)D may influence fat metabolism but its impact on steatosis might not be evident at an early stage. In contrast, fibrosis, as a later-stage pathology, might be more susceptible to 25(OH)D deficiency, particularly due to its anti-inflammatory effects. The other is that steatosis and fibrosis represent different phases of liver disease progression. Steatosis is often reversible, while fibrosis is harder to reverse. The role of 25(OH)D could be more critical in the progression of fibrosis than in the initial fat accumulation stage. Therefore, further exploration of the relationship between 25(OH)D and liver fibrosis is warranted.

In addition, the association between 25(OH)D and fibrosis was different in male and female individuals. Sex hormones have a potential influence on both T2DM and liver steatosis. Previous studies have found that the influences of 25(OH)D deficiency and supplementation on lipid and glucose metabolite changes were sex-specific (38). The results of a meta-analysis indicated that female T2DM patients may have a higher risk of 25(OH)D deficiency (15). Sex hormones are also involved in liver fat accumulation and are closely associated with NAFLD (39, 40). Furthermore, a significant inverse association between sex hormone-binding globulin (SHBG) and the risk of NAFLD has been reported (41). 25(OH)D and sex hormones have complex interactions. Vitamin D metabolites are involved in the regulation of sex hormones and their interaction plays an important role in the regulation of lipid metabolism and obesity (42). In addition, the association between 25(OH)D and sex hormones was reported to be different in different genders and women with different menopausal statuses (43). For example, 25(OH)D levels were found to be significantly associated with testosterone levels while the association between 25(OH)D and sex hormones in women was inconclusive (42). Testosterone has been linked to increased liver fibrosis, as it may promote fibrogenesis through mechanisms such as enhancing collagen synthesis and activation of hepatic stellate cells. Conversely, estrogen has protective effects against liver fibrosis, potentially promoting apoptosis of hepatic stellate cells and inhibiting collagen production. The contrasting effects of these hormones can thus create a gender-based difference in how 25(OH)D influences liver fibrosis. Additionally, SHBG levels, which are influenced by hormonal status, can affect bioavailable sex hormones and thereby modulate their actions on liver tissue. Lower SHBG levels in men may lead to higher free testosterone levels, resulting in an increased risk of liver fibrosis. Due to the complex interaction between 25(OH)D and sex hormones, as well as their involvement in liver steatosis, the influence of 25(OH)D therefore may be affected by sex. It has been suggested that the multiple mechanisms through which 25(OH)D participates in the regulation of NAFLD development, including insulin sensitivity, lipid metabolism and inflammation, may be affected by individual sex hormone levels (44, 45). Previous studies have suggested that sex differences affected the relationship between 25(OH)D deficiency and NAFLD (23). Furthermore, menopause status is among the confounding factors that influence the association between NAFLD and T2DM (46). The association between sex and liver fibrosis may vary in women before and after menopause (47). Therefore, the risk of 25(OH)D deficiency, as well as its influence on the development of liver fibrosis, may be different in male and female T2DM patients. Clinically, these sex-specific differences can be attributed to several key factors and underlying mechanisms. For women, the hormonal fluctuations post-menopause lead to changes in lipid metabolism and an increased risk of NAFLD. The decline in estrogen levels may alter the metabolism of 25(OH)D, affecting its bioavailability and overall impact on liver health. Additionally, 25(OH)D plays a crucial role in anti-inflammatory and antifibrotic processes, and its deficiency might exacerbate liver inflammation and fibrosis progression. In contrast, men typically exhibit higher levels of visceral adiposity, which is associated with more severe liver fibrosis. This distinct fat distribution and the differential impact on insulin sensitivity might further affect the risk of NAFLD and subsequent fibrosis in male patients with T2DM. Moreover, sex-specific differences in body composition, metabolic rate, and liver enzyme functions could also contribute to the varying effects of 25(OH)D deficiency on liver fibrosis. Understanding these distinctions is crucial for developing personalized therapeutic strategies and improving the overall prognosis for patients with T2DM. The interplay between sex, menopause status, hormonal environment, and metabolic differences underscores the need for a differentiated approach in managing 25(OH)D levels and their implications for liver health in male and female patients with T2DM.

The study is subject to certain limitations. Due to the complexity of the potential risk factors, limited sample size, and wide distribution of some tested parameters, the validities of differences and correlations detected need to be further confirmed in a larger population. Furthermore, interactions among the tested factors need to be further addressed. The influence of 25(OH)D levels on liver fibrosis may be clarified in a stratified analysis of 25(OH)D deficiency, although this may depend on the normal range cutoff. Nevertheless, the results from this analysis did raise an interesting finding that not only was 25(OH)D associated with liver fibrosis despite subtle changes in serum levels, but its influence closely interacted with sex. Therefore, further confirmation and study of the underlying mechanisms are warranted.

In conclusion, a cross-sectional study in patients with T2DM showed that serum 25(OH)D level is strongly associated with liver fibrosis, and this relationship was more pronounced in male patients.

## Data Availability

The data underlying this article is available from the corresponding author under reasonable request.

## References

[1] Castera L Laouenan C Vallet-Pichard A Vidal-Trecan T Manchon P Paradis V. High prevalence of NASH and advanced fibrosis in type 2 diabetes: A prospective study of 330 outpatients undergoing liver biopsies for elevated ALT, using a low threshold. Diabetes Care. (2023) 46(7):1354–62. doi: 10.2337/dc22-2048 37043830

[2] Furuta K Tang X Islam S Tapia A Chen ZB Ibrahim SH. Endotheliopathy in the metabolic syndrome: Mechanisms and clinical implications. Pharmacol Ther. (2023) 244:108372. doi: 10.1016/j.pharmthera.2023.108372 36894027 PMC10084912

[3] Eslam M El-Serag HB Francque S Sarin SK Wei L Bugianesi E. Metabolic (dysfunction)-associated fatty liver disease in individuals of normal weight. Nat Rev Gastroenterol hepatology. (2022) 19(10):638–51. doi: 10.1038/s41575-022-00635-5 35710982

[4] Ajmera V Cepin S Tesfai K Hofflich H Cadman K Lopez S. A prospective study on the prevalence of NAFLD, advanced fibrosis, cirrhosis and hepatocellular carcinoma in people with type 2 diabetes. J hepatology. (2023) 78(3):471–8. doi: 10.1016/j.jhep.2022.11.010 PMC9975077 36410554

[5] LoMonaco R Godinez Leiva E Bril F Shrestha S Mansour L Budd J. Advanced liver fibrosis is common in patients with type 2 diabetes followed in the outpatient setting: the need for systematic screening. Diabetes Care. (2021) 44(2):399–406. doi: 10.2337/dc20-1997 33355256 PMC7818321

[6] Ciardullo S Monti T Perseghin G. High prevalence of advanced liver fibrosis assessed by transient elastography among U.S. Adults with type 2 diabetes. Diabetes Care. (2021) 44:519–25. doi: 10.2337/dc20-1778 33303638

[7] Luukkonen PK Qadri S Ahlholm N Porthan K Mannisto V Sammalkorpi H. Distinct contributions of metabolic dysfunction and genetic risk factors in the pathogenesis of non-alcoholic fatty liver disease. J Hepatology. (2022) 76(3):526–35. doi: 10.1016/j.jhep.2021.10.013 PMC8852745 34710482

[8] Stefan N Cusi K. A global view of the interplay between non-alcoholic fatty liver disease and diabetes. Lancet Diabetes Endocrinology. (2022) 10:284–96. doi: 10.1016/S2213-8587(22)00003-1 35183303

[9] van Valkengoed IGM Argmann C Ghauharali-van der Vlugt K Aerts J Brewster LM Peters RJG. Ethnic differences in metabolite signatures and type 2 diabetes: a nested case-control analysis among people of South Asian, African and European origin. Nutr Diabetes. (2017) 7(12):300. doi: 10.1038/s41387-017-0003-z 29259157 PMC5865542

[10] Hatano Y VanWagner LB Carnethon MR Bancks MP Carson AP Lloyd-Jones DM. Racial difference in the association between non-alcoholic fatty liver disease and incident type 2 diabetes: findings from the CARDIA study. Diabetologia. (2023) 66(7):1235–46. doi: 10.1007/s00125-023-05903-w PMC10286118 36941389

[11] Fleet JC. The role of vitamin D in the endocrinology controlling calcium homeostasis. Mol Cell Endocrinology. (2017) 453:36–45. doi: 10.1016/j.mce.2017.04.008 PMC5529228 28400273

[12] Aranow C. Vitamin D and the immune system. J Invest medicine: Off Publ Am Fed Clin Res. (2011) 59:881–6. doi: 10.2310/JIM.0b013e31821b8755 PMC3166406 21527855

[13] Oh J Riek AE Bauerle KT Dusso A McNerney KP Barve RA. Embryonic vitamin D deficiency programs hematopoietic stem cells to induce type 2 diabetes. Nat Commun. (2023) 14(1):3278. doi: 10.1038/s41467-023-38849-z 37311757 PMC10264405

[14] Gallagher JC Rosen CJ. Vitamin D: 100 years of discoveries, yet controversy continues. Lancet Diabetes endocrinology. (2023) 11:362–74. doi: 10.1016/S2213-8587(23)00060-8 37004709

[15] Taderegew MM Woldeamanuel GG Wondie A Getawey A Abegaz AN Adane F. Vitamin D deficiency and its associated factors among patients with type 2 diabetes mellitus: a systematic review and meta-analysis. BMJ Open. (2023) 13:e075607. doi: 10.1136/bmjopen-2023-075607 PMC10565281 37798019

[16] Christides T. Vitamin D and risk of type 2 diabetes. BMJ (Clinical Res ed). (2022) 377:o1166. doi: 10.1136/bmj.o1166 35613716

[17] Ahmed LHM Butler AE Dargham SR Latif A Robay A Chidiac OM. Association of vitamin D(2) and D(3) with type 2 diabetes complications. BMC endocrine Disord. (2020) 20(1):65. doi: 10.1186/s12902-020-00549-w PMC7227254 32414363

[18] Chen X Wan Z Geng T Zhu K Li R Lu Q. Vitamin D status, vitamin D receptor polymorphisms, and risk of microvascular complications among individuals with type 2 diabetes: A prospective study. Diabetes Care. (2023) 46(2):270–7. doi: 10.2337/dc22-0513 36169213

[19] Kawahara T Suzuki G Mizuno S Inazu T Kasagi F Kawahara C. Effect of active vitamin D treatment on development of type 2 diabetes: DPVD randomised controlled trial in Japanese population. BMJ (Clinical Res ed). (2022) 377:e066222. doi: 10.1136/bmj-2021-066222 PMC9131780 35613725

[20] Geng T Lu Q Wan Z. Association of serum 25-hydroxyvitamin D concentrations with risk of dementia among individuals with type 2 diabetes: A cohort study in the UK Biobank. PloS Med. (2022) 19:e1003906. doi: 10.1371/journal.pmed.1003906 35025861 PMC8797194

[21] Harris E. Meta-analysis: vitamin D therapy reduced type 2 diabetes. Jama. (2023) 329:703. doi: 10.1001/jama.2023.1550 36790836

[22] Yuan S Larsson SC. Inverse association between serum 25-hydroxyvitamin D and nonalcoholic fatty liver disease. Clin Gastroenterol hepatology: Off Clin Pract J Am Gastroenterological Assoc. (2023) 21:398–405.e394. doi: 10.1016/j.cgh.2022.01.021 35101633

[23] Sharifi N Amani R. Vitamin D supplementation and non-alcoholic fatty liver disease: A critical and systematic review of clinical trials. Crit Rev Food Sci Nutr. (2019) 59:693–703. doi: 10.1080/10408398.2017.1389693 29035092

[24] Bjelakovic G Nikolova D Bjelakovic M Gluud C. Vitamin D supplementation for chronic liver diseases in adults. Cochrane Database systematic Rev. (2017) 11:Cd011564. doi: 10.1002/14651858.CD011564.pub2 PMC6485973 29099543

[25] Kim HS Rotundo L Feurdean M. Vitamin D's role in non-alcoholic fatty liver disease. Am J gastroenterology. (2017) 112:806–7. doi: 10.1038/ajg.2016.602 28469226

[26] Patel YA Henao R Moylan CA Guy CD Piercy DL Diehl AM. Vitamin D is not associated with severity in NAFLD: results of a paired clinical and gene expression profile analysis. Am J gastroenterology. (2016) 111(11):1591–8. doi: 10.1038/ajg.2016.406 PMC5331905 27644736

[27] Zhou Q Wang Y Wang J Liu Y Qi D Yao W. Prevalence and risk factor analysis for the nonalcoholic fatty liver disease in patients with type 2 diabetes mellitus. Medicine. (2021) 100(10):e24940. doi: 10.1097/MD.0000000000024940 33725855 PMC7969325

[28] Guan C Fu S Zhen D Yang K An J Wang Y. Metabolic (Dysfunction)-associated fatty liver disease in Chinese patients with type 2 diabetes from a subcenter of the national metabolic management center. J Diabetes Res. (2022) 2022:8429847. doi: 10.1155/2022/8429847 35127953 PMC8816602

[29] Fabbrini E Sullivan S Klein S. Obesity and nonalcoholic fatty liver disease: biochemical, metabolic, and clinical implications. Hepatol (Baltimore Md). (2010) 51:679–89. doi: 10.1002/hep.23280 PMC3575093 20041406

[30] Mokdad AH Ford ES Bowman BA Dietz WH Vinicor F Bales VS. Prevalence of obesity, diabetes, and obesity-related health risk factors, 2001. Jama. (2003) 289(1):76–9. doi: 10.1001/jama.289.1.76 12503980

[31] Li Y Qin M Zhong W Liu C Deng G Yang M. RAGE promotes dysregulation of iron and lipid metabolism in alcoholic liver disease. Redox Biol. (2023) 59:102559. doi: 10.1016/j.redox.2022.102559 36502724 PMC9758571

[32] Utzschneider KM Kahn SE. The role of insulin resistance in nonalcoholic fatty liver disease. J Clin Endocrinol Metab. (2006) 91:4753–61. doi: 10.1210/jc.2006-0587 16968800

[33] Stefan N Schick F Birkenfeld AL Häring HU White MF. The role of hepatokines in NAFLD. Cell Metab. (2023) 35:236–52. doi: 10.1016/j.cmet.2023.01.006 PMC10157895 36754018

[34] Gong L Wei F Gonzalez FJ Li G. Hepatic fibrosis: Targeting peroxisome proliferator-activated receptor alpha from mechanism to medicines. Hepatol (Baltimore Md). (2023) 78:1625–53. doi: 10.1097/HEP.0000000000000182 PMC10681123 36626642

[35] Targher G Bertolini L Padovani R Rodella S Tessari R Zenari L. Prevalence of nonalcoholic fatty liver disease and its association with cardiovascular disease among type 2 diabetic patients. Diabetes Care. (2007) 30:1212–8. doi: 10.2337/dc06-2247 17277038

[36] Younossi ZM Koenig AB Abdelatif D Fazel Y Henry L Wymer M. Global epidemiology of nonalcoholic fatty liver disease-Meta-analytic assessment of prevalence, incidence, and outcomes. Hepatol (Baltimore Md). (2016) 64:73–84. doi: 10.1002/hep.28431 26707365

[37] Yamane R Yoshioka K Hayashi K Shimizu Y Ito Y Matsushita K. Prevalence of nonalcoholic fatty liver disease and its association with age in patients with type 2 diabetes mellitus. World J hepatology. (2022) 14(16):1226–34. doi: 10.4254/wjh.v14.i6.1226 PMC9258257 35978658

[38] Hu T Ren L Li H An Z. Effects of Vitamin D supplementation or deficiency on metabolic phenotypes in mice of different sexes. J Steroid Biochem Mol Biol. (2023) 229:106250. doi: 10.1016/j.jsbmb.2023.106250 36708934

[39] Waxman DJ Kineman RD. Sex matters in liver fat regulation. Sci (New York NY). (2022) 378:252–3. doi: 10.1126/science.ade7614 36264790

[40] Kasarinaite A Sinton M Saunders PTK Hay DC. The influence of sex hormones in liver function and disease. Cells. (2023) 12. doi: 10.3390/cells12121604 PMC10296738 37371074

[41] Kim D Manikat R Cholankeril G Ahmed A. Endogenous sex hormones and nonalcoholic fatty liver disease in US adults. Liver international: Off J Int Assoc Study Liver. (2023). doi: 10.1111/liv.15786 38010926

[42] Zhao D Ouyang P de Boer IH Lutsey PL Farag YM Guallar E. Serum vitamin D and sex hormones levels in men and women: The Multi-Ethnic Study of Atherosclerosis (MESA). Maturitas. (2017) 96:95–102. doi: 10.1016/j.maturitas.2016.11.017 28041602 PMC5218632

[43] Wang N Zhai H Zhu C Li Q Han B Chen Y. Combined association of vitamin D and sex hormone binding globulin with nonalcoholic fatty liver disease in men and postmenopausal women: A cross-sectional study. Medicine. (2016) 95(4):e2621. doi: 10.1097/MD.0000000000002621 26825918 PMC5291588

[44] Javed Z Papageorgiou M Deshmukh H Kilpatrick ES Mann V Corless L. A randomized, controlled trial of vitamin D supplementation on cardiovascular risk factors, hormones, and liver markers in women with polycystic ovary syndrome. Nutrients. (2019) 11(1). doi: 10.3390/nu11010188 PMC6356309 30658483

[45] Du T Xiang L Zhang J Yang C Zhao W Li J. Vitamin D improves hepatic steatosis in NAFLD via regulation of fatty acid uptake and β-oxidation. Front endocrinology. (2023) 14(8):1138078. doi: 10.3389/fendo.2023.1138078 PMC10074590 37033263

[46] Kim Y Chang Y Ryu S Wild SH Byrne CD. NAFLD improves risk prediction of type 2 diabetes: with effect modification by sex and menopausal status. Hepatol (Baltimore Md). (2022) 76:1755–65. doi: 10.1002/hep.32560 35514152

[47] Burra P Bizzaro D Gonta A Shalaby S Gambato M Morelli MC. Clinical impact of sexual dimorphism in non-alcoholic fatty liver disease (NAFLD) and non-alcoholic steatohepatitis (NASH). Liver international: Off J Int Assoc Study Liver. (2021) 41:1713–33. doi: 10.1111/liv.14943 33982400

